# De-Identification of Facial Features in Magnetic Resonance Images: Software Development Using Deep Learning Technology

**DOI:** 10.2196/22739

**Published:** 2020-12-10

**Authors:** Yeon Uk Jeong, Soyoung Yoo, Young-Hak Kim, Woo Hyun Shim

**Affiliations:** 1 Department of Medical Science Asan Medical Institute of Convergence Science and Technology, Asan Medical Center University of Ulsan College of Medicine Seoul Republic of Korea; 2 Human Research Protection Center Asan Institute of Life Sciences, Asan Medical Center University of Ulsan College of Medicine Seoul Republic of Korea; 3 Division of Cardiology Department of Internal Medicine, Asan Medical Center University of Ulsan College of Medicine Seoul Republic of Korea; 4 Department of Information Medicine Asan Medical Center University of Ulsan College of Medicine Seoul Republic of Korea; 5 Department of Radiology Asan Medical Center University of Ulsan College of Medicine Seoul Republic of Korea

**Keywords:** de-identification, privacy protection, personal information protection, medical image, deep learning, facial feature detection, HIPAA, GDPR

## Abstract

**Background:**

High-resolution medical images that include facial regions can be used to recognize the subject’s face when reconstructing 3-dimensional (3D)-rendered images from 2-dimensional (2D) sequential images, which might constitute a risk of infringement of personal information when sharing data. According to the Health Insurance Portability and Accountability Act (HIPAA) privacy rules, full-face photographic images and any comparable image are direct identifiers and considered as protected health information. Moreover, the General Data Protection Regulation (GDPR) categorizes facial images as biometric data and stipulates that special restrictions should be placed on the processing of biometric data.

**Objective:**

This study aimed to develop software that can remove the header information from Digital Imaging and Communications in Medicine (DICOM) format files and facial features (eyes, nose, and ears) at the 2D sliced-image level to anonymize personal information in medical images.

**Methods:**

A total of 240 cranial magnetic resonance (MR) images were used to train the deep learning model (144, 48, and 48 for the training, validation, and test sets, respectively, from the Alzheimer's Disease Neuroimaging Initiative [ADNI] database). To overcome the small sample size problem, we used a data augmentation technique to create 576 images per epoch. We used attention-gated U-net for the basic structure of our deep learning model. To validate the performance of the software, we adapted an external test set comprising 100 cranial MR images from the Open Access Series of Imaging Studies (OASIS) database.

**Results:**

The facial features (eyes, nose, and ears) were successfully detected and anonymized in both test sets (48 from ADNI and 100 from OASIS). Each result was manually validated in both the 2D image plane and the 3D-rendered images. Furthermore, the ADNI test set was verified using Microsoft Azure's face recognition artificial intelligence service. By adding a user interface, we developed and distributed (via GitHub) software named “Deface program” for medical images as an open-source project.

**Conclusions:**

We developed deep learning–based software for the anonymization of MR images that distorts the eyes, nose, and ears to prevent facial identification of the subject in reconstructed 3D images. It could be used to share medical big data for secondary research while making both data providers and recipients compliant with the relevant privacy regulations.

## Introduction

It is becoming important to handle and share big data in the health care field, and accordingly, there is a big trend to share and protect individual patient data for secondary research [1-3]. To utilize big data, data anonymization is necessary so as not to violate laws for personal privacy such as those stipulated by the Health Insurance Portability and Accountability Act (HIPAA) in the United States and General Data Protection Regulation (GDPR) in Europe [4,5]. There is a trade-off between data usability and privacy protection. Nevertheless, sufficient administrative and technical measures for previously collected information in accordance with personal information protection regulations are necessary when using the information secondarily without consent.

High-resolution magnetic resonance (MR) images of the head risk exposing a subject’s face, which can be regarded at the level of photography by facial reconstruction [6]. According to HIPAA's privacy rules, full-face photographic images and any comparable images are considered to be protected health information (Multimedia Appendix 1). Budin et al [7] tested human observer recognition of 3-dimensional (3D)-rendered MR images and reported that the likelihood of correctly matching a 3D-rendered face image with a portrait of that person is higher than random guessing. Additionally, anyone can reproduce the 3D facial image from head MR images through 3D volume rendering using freeware. Therefore, it is necessary to anonymize medical images that include the face.

Facial image anonymization is not fully conducted in public medical image repositories, while some public databases even provide the original images. For example, the Alzheimer's Disease Neuroimaging Initiative (ADNI) [8] and Open Access Series of Imaging Studies (OASIS) [9] usually anonymize only metadata, while the original MR images are shared in a nonanonymized form. Anonymizing only the metadata from the medical image is not sufficient to prevent identification from the remaining medical images after removing the metadata, and existing anonymizing software is rarely used to prevent the possibility of recognition due to concerns over the deterioration of the brain image quality [10].

Previous approaches to anonymizing faces in medical images usually remove the entire facial region using a voxel classifier and mask the brain to preserve the brain image using a skull stripping technique or a convex hull [11,12]. However, since using a voxel classifier and skull stripping can be affected by variation in the characteristics of the MR images, they can produce unexpected results from heterogeneous MR image data [13]. In addition, the solution of cutting off the face has the limitation of information loss concerning the eye orbits, nasal cavity, and other underlying structures [6]. In anonymization work for medical image sharing, consistent processing of heterogeneous data and minimizing data loss will help researchers using secondary data.

The aim of this study was to develop software that can selectively distort the eyes, nose, and ears, which are the main factors for identifying a face, and make a robust anonymization algorithm that can be used on various MR images.

## Methods

### Defacing Process Overview

**Figure 1:**
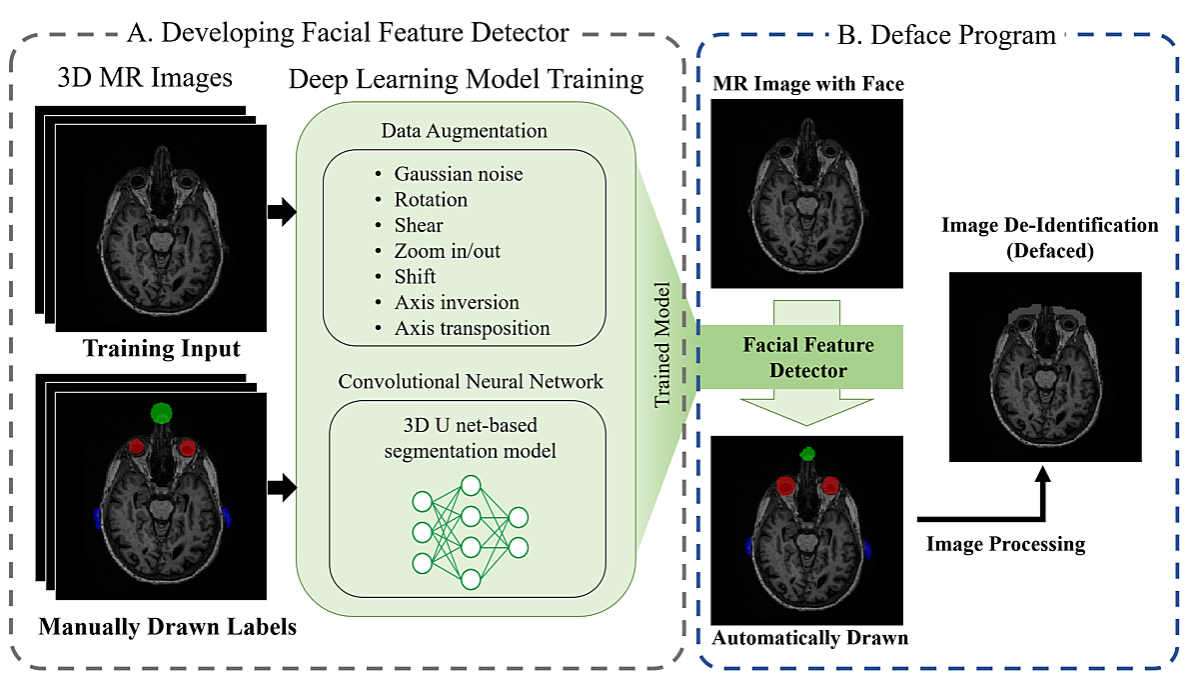
Process of (A) developing the facial feature detector, which is a deep learning model that can detect the eyes, nose, and ears in 3-dimensional (3D) magnetic resonance (MR) images, and (B) distorting the facial features in nonanonymized cranial MR images.

Figure 1 schematically illustrates our Deface program development process (Figure 1A) and an application example (Figure 1B). We created a deep learning model that learns the labels of the eyes, nose, and ears. The training set consisted of 3D cranial MR images and manually marked regions corresponding to each MR image. We implemented data augmentation to increase the diversity of the training data. The deep learning model was developed based on a 3D convolutional neural network. The trained model, called a “facial feature detector,” can detect the eyes, nose, and ears in a 3D MR image. After the regions of the facial features have been obtained from a nonanonymous 3D MR image through the facial feature detector, the regions are anonymized according to each characteristic.

### Image Acquisition

The Neuroimaging Informatics Technology Initiative (NIFTI) and Digital Imaging and Communications in Medicine (DICOM) formats of MR imaging (MRI) files were collected from the ADNI database (Magnetization Prepared RApid Gradient Echo [MPRAGE] scans; voxel size: 1.0 x 1.0 x 1.2 mm; inplane resolution: 1.0 x 1.0 mm^2^; interslice spacing: 1.2 mm; field of view [FOV]: 240 x 256 x 160 mm). A total of 240 NIFTI format files were used in the creation of the deep learning model: 144, 48, and 48 for the training, validation, and test sets, respectively.

Other NIFTI-format MRI files were collected from the OASIS-3 database for use as the external test set. The 100 MR images differed in orientation, resolution, and intensity from those in the ADNI data (MPRAGE scans; voxel size: 1.0 x 1.0 x 1.0 mm; FOV: 176 x 256 x 256 mm).

### Labeling

In general, supervised learning requires pairs consisting of the input object and the desired output value. In this study, the input object is a 3D cranial MR image, and the output values are regions containing the eyes, nose, or ears (the facial features). We manually drew labels that were the same as the desired output values in all of the ADNI and 20 OASIS-3 images using the AFNI program [14]. In Figure 1A, the manually drawn labels show the eyes (red) and nose (green), which are marked as spherical shapes at the corresponding positions, and the ears (blue), which are marked as the auricle regions. Each center point of the eyes and nose area was labeled in the form of a sphere. Since ears have different sizes and shapes for each person, only the auricle of the ear was segmented and labeled.

### Data Augmentation

Three image augmentations were performed per 1 image in the training set. The augmented images were randomly transformed and then used for model training. As a result, 576 images per epoch were trained. Data augmentation was performed by filtering Gaussian noise, rotating from –15° to +15° around each axis in the image, randomly flipping each axis, randomly transposing between the axes, shifting each axis from 0 to 0.10, shearing each axis from 0 to 0.20, and resizing the image from 0.90 to 1.10 times the original size. After executing 1 image augmentation per original image, the validation set was validated for a total of 96 images per epoch.

### The Deep Learning Algorithm

The deep learning model was trained with the manually labeled data. We created a deep learning model that can generate labels similar to manually drawn labels on the regions of the eyes, nose, and ears from cranial MR image input. The basic structure of our deep learning model is attention-gated U-net [15]. The detailed structure of our model can be found in Multimedia Appendix 2.

### Metric and Loss Function

In machine learning, the “loss” or “error” variable is set to achieve the goals through the training of the model. In addition, the “metric” variable indicates how much we have achieved the goals through the model training. A machine learning model has metrics to indicate the achievement rate and is trained to reduce loss.

In this study, the metric to determine whether the model can make labels similar to the manually drawn labels is the Dice coefficient, which is double the area of overlap divided by the total number of pixels or voxels in both images: It returns 1 if the predicted regions of the model exactly match the correct answers from the labels and 0 if the regions do not overlap. When the region of the label is *Y* and the region predicted by the trained model is *X*, the Dice coefficient can be represented by:


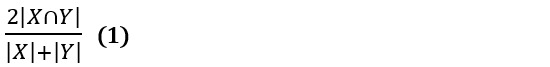


This can also be expressed as:


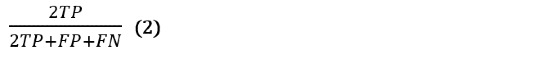


where *TP* is the number of true positives, *FP* is the number of false positives, and *FN* is the number of false negatives.

The loss function in our model was: 1 – the Dice coefficient + 0.1 × categorical cross-entropy. Categorical cross-entropy is the loss function mainly used in multiclass classification, and it induces our model to learn to distinguish whether a specific pixel is from the eye, nose, ear, or another area. This model computes the loss function between the correct answer labels and the predictive labels and is trained in the direction of loss reduction (toward zero).

The model calculated Dice coefficients for 96 images in the validation set for each epoch. After 5 epochs at the highest metric score, learning was stopped when there was no further improvement.

### Image Processing

Here, we describe the process of image anonymization based on the output of the facial feature detector. The deep learning model was trained by identifying the eyes, nose, and ears (5 regions), after which the program proceeded with the image anonymization process.

Identification of the eyes, nose, and ears was automatically conducted on different images according to each feature by the deep learning algorithm. The detection region for the eyes is a spherical area covering the eyeball and the skin around the eye. The process of anonymizing the surface of the eye consists of 2 steps. First, based on the detection regions for the eyes, 2 boxes capable of covering the periocular area (the skin around the eyes) are formed. Second, the contour of the face surface was obtained within the range of the boxes, and a range of ±2 voxels along each axis from that surface was modified to the same intensity value. The nose was processed by removing the image and setting the intensity of the voxels to 0 in the area where the binding box for the detected region was doubled to each side. The detection region for the ears is the protruding part called the auricle. For the anonymization of the ears, random values were assigned to each voxel of the detection regions of the ears, and those values were generated in the noise range of the air in the MR image.

In the case of the medical images in DICOM format, it is necessary to anonymize the personal information in the header, and so we carried this out on the 20 DICOM headers using the Deface program (the DICOM headers are listed in Multimedia Appendix 3). The list was selected based on the HIPAA safe harbor provision [16].

## Results

In the 23rd epoch, the average Dice coefficient of the validation set was the highest at 0.821. In the 28th epoch, the training of the model was stopped because the Dice score of the validation set did not improve. The average Dice score of 576 images trained over 23 epochs was 0.801. The average Dice scores using the test sets comprising 48 ADNI and 20 OASIS-3 images were 0.859 and 0.794, respectively. The Deface program was applied to the ADNI data, but anonymization was performed on the OASIS-3 data without any additional manipulation.

Figure 1B shows the process of distorting a sample nonanonymized cranial MR image. Three axial views of the cross-sectional MR image were obtained from a representative image in the ADNI test set. The first is the nonanonymized cranial MR image, the second is an MR image with the detection regions (the labels of the eyes, nose, and ears predicted by the facial feature detector) as output for the facial feature detector, and the third is the final anonymized image based on the detection regions (red marks denoting the eyes, green marks denoting the nose, and blue marks denoting the ears). It took 177.91 seconds to save the detection region pictures and distorted MR images as NIFTI format files from 48 images of the ADNI test set. The image was distorted according to the characteristics of each facial feature. The 3D box space containing the entire volume of the nose was removed. The eyes were covered with similar brightness intensity on the surface. For the ears, the detection regions were replaced by space with noise.

**Figure 2:**
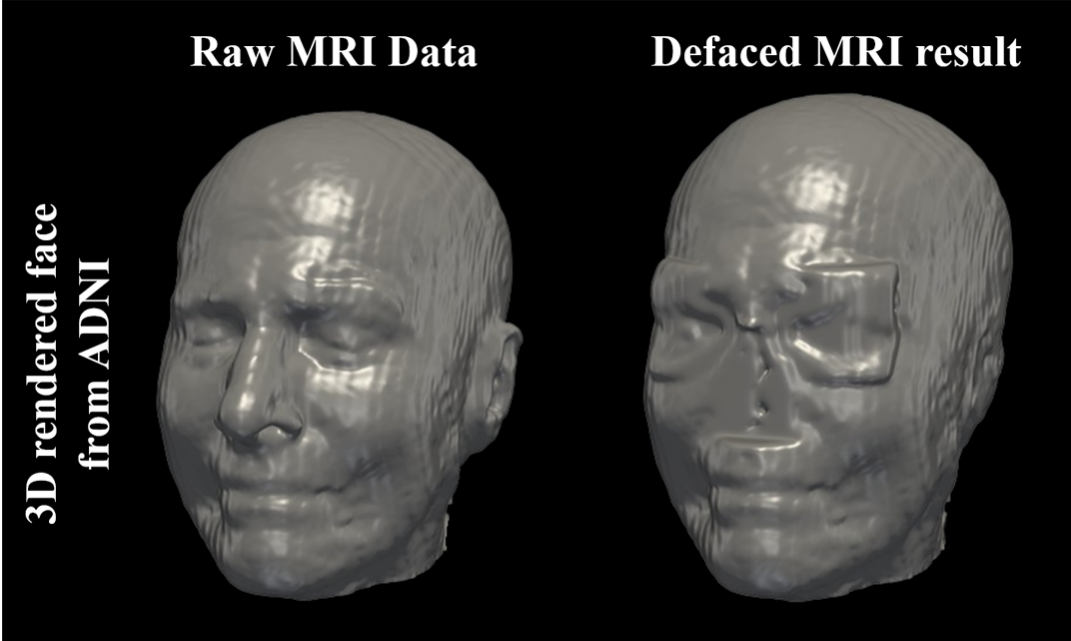
3-dimensional (3D) volume rendering of magnetic resonance images (MRI), showing the raw and distorted images from the Alzheimer's Disease Neuroimaging Initiative (ADNI) .

We applied the Deface program to 48 ADNI images and 100 OASIS-3 images as the test sets and then confirmed the accuracy of distorting the facial features in the 3D reconstructions of the face (Figure 2 shows the 3D volume-rendered images). Since face reconstruction is in violation of the OASIS data use terms, OASIS data were not included in the figure. A sample image was selected from the ADNI test sets, and we compared the before and after anonymization. As shown in Figure 2, the facial features clearly identifiable in the 3D images beforehand are distorted after processing: The auricle and nose have disappeared, and the eyes appear blurry.

The Deface program was used to validate the de-identification performance by Microsoft Azure’s facial recognition artificial intelligence service (Face detection_01 model) [17]. We found that all 48 reconstructed face images from the ADNI test set were de-identified. Although 46 unmodified images were recognized as faces and location information of face landmarks was derived, the faces in all 48 defaced images were not recognized. The other 2 unmodified images failed the face recognition process because they were noisy or parts of the face were cropped. The result of the face detection service for 1 representative image of the ADNI test set can be found in Multimedia Appendix 4.

## Discussion

### Principal Findings

In this study, we developed a program that can recognize the eyes, nose, and ears in MR images by applying artificial intelligence, after which they were blurred. We implemented the facial feature detector based on the 3D U-net deep learning model to automatically detect the eyes, nose, and ears. The reason for the development of this anonymization program is that 3D facial reconstruction of high-resolution MRI can show an individual’s similarity to a facial photograph [6,7], which contravenes the rules for protecting personal information required by regulating bodies such as HIPAA. Anonymization is required for the sharing of medical image data so as not to infringe on the personal information rules. However, distorting images is disadvantageous for secondary research due to the loss of information, but too little distorting leads to the possibility of recognition [10]. We attempted to anonymize the face while minimizing the loss of information by modifying only the surface of the eyes, nose, and ears in cranial MR images. In addition, in the case of the DICOM format, a function to remove text including personal information that can be obtained from the header was added. We released the source code to GitHub [18].

Figure 1A shows the process of developing the facial feature detector. The cranial MR images and manually marked facial features (eyes, nose, and ears) were used as the training set. We drew different labels for each facial feature for the manually drawn labels. Although the eyes and nose can be specified in a range of only the central location information, the shape of the ear varies relatively widely among people. Furthermore, because the ears are adjacent to the brain, images of the brain can be obscured during the image distortion Therefore, only the segmented regions of the auricle were used as labels so that the program did not select regions other than the ear.

Although the training data for the deep learning model comprised 144 images from ADNI, we introduced data augmentation to achieve robust performance in other MRI standards (Figure 1A). The training set was augmented via various techniques so that the facial feature detector could show robust performance even with unknown data. We evaluated OASIS-3 data in which the adjustment, orientation, FOV, resolution, and intensity histograms were completely different from the ADNI data in the training set. We confirmed that the facial features were distorted in 100 OASIS-3 images by the MRI viewer. Labels were manually drawn on 20 OASIS-3 images, and our facial feature detector worked well, with an average Dice coefficient of 0.794. This has the potential to assist in the construction of anonymous big data with different MRI standards collected from multiple institutions.

We applied different processes to blur each facial feature location. The eyes are close to the frontal lobe, so they were distorted only along the surface. The intensity of the pixels was converted to a value similar to the surface of the skin to make it appear on the surface when 3D rendering. Since the nose is usually the most protuberant part of the face, the area that covers the entire range of the nose was deleted to make it impossible to infer the original shape of the nose. The 3D box space containing the entire volume of the nose was removed to prevent recognition via the nose shape. The ears were only segmented by the facial feature detector, so only the corresponding regions were distorted to preserve the brain image. If regions such as the shape of the ears are simply removed, the shape of the ears may be revealed by the noise from air in the MR image. We reduced the possibility of recognition by replacing the ear regions with generated random values within the air noise range of the input MR image.

3D facial reconstruction of high-resolution MRI can be generated by a freeware MRI viewer [19]. Moreover, the faces of patients in MR images from publicly available data can be revealed (Figure 2). As the OASIS-3 images are smoother than the ADNI images, they can be reconstructed with a clearer face image in the case of high-resolution MRI. However, we showed that the face could be distorted in the 3D-rendered image after applying our Deface program. Since the image was preserved except for the user-designated facial features, researchers can obtain the necessary information from MRI images without revealing the patient’s identity.

### Comparison With Prior Work

Previous studies have applied techniques to remove the entire face regions, and the evaluation of anonymization was via direct human observation of face landmarks [7,11,12]. In another technique, the Human Connectome Project [20], a public repository of MRI images, conducted distorting by modifying a certain thickness of the facial surface [13]. We distorted the ears in addition to the face surface, with options to blur the eyes, nose, and ears separately, as may be required when conducting secondary research. The images with eyes, nose, and ears anonymized were verified by applying a face recognition tool. Furthermore, while previous studies have applied algorithms to process single MRI datasets, our Deface program was tested on 2 different MRI datasets to improve compatibility.

### Limitations

Among the facial features, wrinkles or the mouth can be identifiers but were not considered in this study. To train the deep learning model, we needed to manually draw labels that mark facial features. We are planning to construct a training dataset that takes into account additional facial features for further study. Once labeled training data comprising any desired facial feature have been constructed, our facial feature detector can evolve through deep learning.

### Conclusions

Patients’ faces can be reconstructed from high-resolution cranial MR images at the photograph level, so there is a risk of infringing the personal information rules prescribed by HIPAA and GDPR when sharing data. Hence, we suggested a method to perceive the facial features in MR images via deep learning technology to specifically blur certain facial features. Users can create anonymization regions that blur the desired parts of the patient’s face (eyes, nose, or ears), which helps provide data for secondary research without violating relevant personal information regulations.

## Appendix

Multimedia Appendix 1 Information Protection Regulations.

Multimedia Appendix 2 Deep learning model structure.

Multimedia Appendix 3 DICOM header with personal information.

Multimedia Appendix 4 Face recognition test.

## Abbreviations

2D: two-dimensional
3D: three-dimensional
ADNI: Alzheimer's Disease Neuroimaging Initiative
DICOM: Digital Imaging and Communications in Medicine
FOV: field of view
GDPR: General Data Protection Regulation
HIPAA: Health Insurance Portability and Accountability Act
MPRAGE: Magnetization Prepared RApid Gradient Echo
MR: magnetic resonance
MRI: magnetic resonance imaging
NIFTI: Neuroimaging Informatics Technology Initiative
OASIS: Open Access Series of Imaging Studies
