# Supplementary material for: De-Identification of Facial Features in Magnetic Resonance Images: Software Development Using Deep Learning Technology
Source: J Med Internet Res. 2020 Dec 10;22(12):e22739. doi: 10.2196/22739 (PMC7759440; doi:10.2196/22739)
Supplement: Multimedia Appendix 1 [file jmir_v22i12e22739_app1.docx]

**Appendix 1.**

## Information Protection Regulations

| Regulation | Details |
| --- | --- |
| HIPAA Privacy Rule [1] | According to the “Safe Harbor Privacy Rule 164.514(b)(2)”, 18 specific identifiers are removed from the records or photographs. The information is then deemed “deidentified” and no longer considered identifiable. Deidentified health information created after this method is no longer protected by the Privacy Rule.  Eighteen identifiers to be removed for deidentification.   1. Name 2. All geographic subdivisions smaller than a state 3. All elements of data (except year) for dates directly related to the individual (date of birth, date of admission, date of discharge, date of death). Also, all ages over 89 years or elements of dates indicative of such age. 4. Telephone numbers 5. Fax numbers 6. Email addresses 7. Social security numbers 8. Medical record numbers 9. Health plan beneficiary numbers 10. Account numbers 11. Certificate/license numbers 12. Vehicle identification or serial numbers including license plate numbers 13. Device identification or serial numbers 14. Web Universal Resource Locators (URLs) 15. Internet Protocol (IP) addresses 16. Biometric identifiers including finger and voice prints 17. Full-face photographs and any comparable images 18. Any other unique identifying number, characteristic or code |
| GDPR [2] | The GDPR makes critical differences between personal data, pseudonymized data, and anonymized data. Facial images are categorized as biometric data under the GDPR and need to be protected. Nevertheless, the further processing of personal data for archiving purposes in the public interest, scientific or historical research purposes or statistical purposes is to be carried out when the controller has assessed the feasibility to fulfil those purposes by processing data which do not permit or no longer permit the identification of data subjects, provided that appropriate safeguards exist (such as, for instance, pseudonymisation of the data), according to the Article 89. Otherwise, the GDPR Recital 26 stipulates that anonymous data is no longer applied to the GRPR. |

## References

1. The HIPAA Privacy Rule. US Department of Health and Human Services website. [cited 2020 Sep 1]; Available from: https://www.hhs.gov/hipaa/for-professionals/privacy/index.html.

2. The General Data Protection Regulation (GDPR). The European Parliament and of the Council. [cited 2020 Sep 1]; Available from: https://ec.europa.eu/info/law/law-topic/data-protection/data-protection-eu_en
