# Supplementary material for: De-Identification of Facial Features in Magnetic Resonance Images: Software Development Using Deep Learning Technology
Source: J Med Internet Res. 2020 Dec 10;22(12):e22739. doi: 10.2196/22739 (PMC7759440; doi:10.2196/22739)
Supplement: Multimedia Appendix 2 [file jmir_v22i12e22739_app2.docx]

**Deep learning model structure**
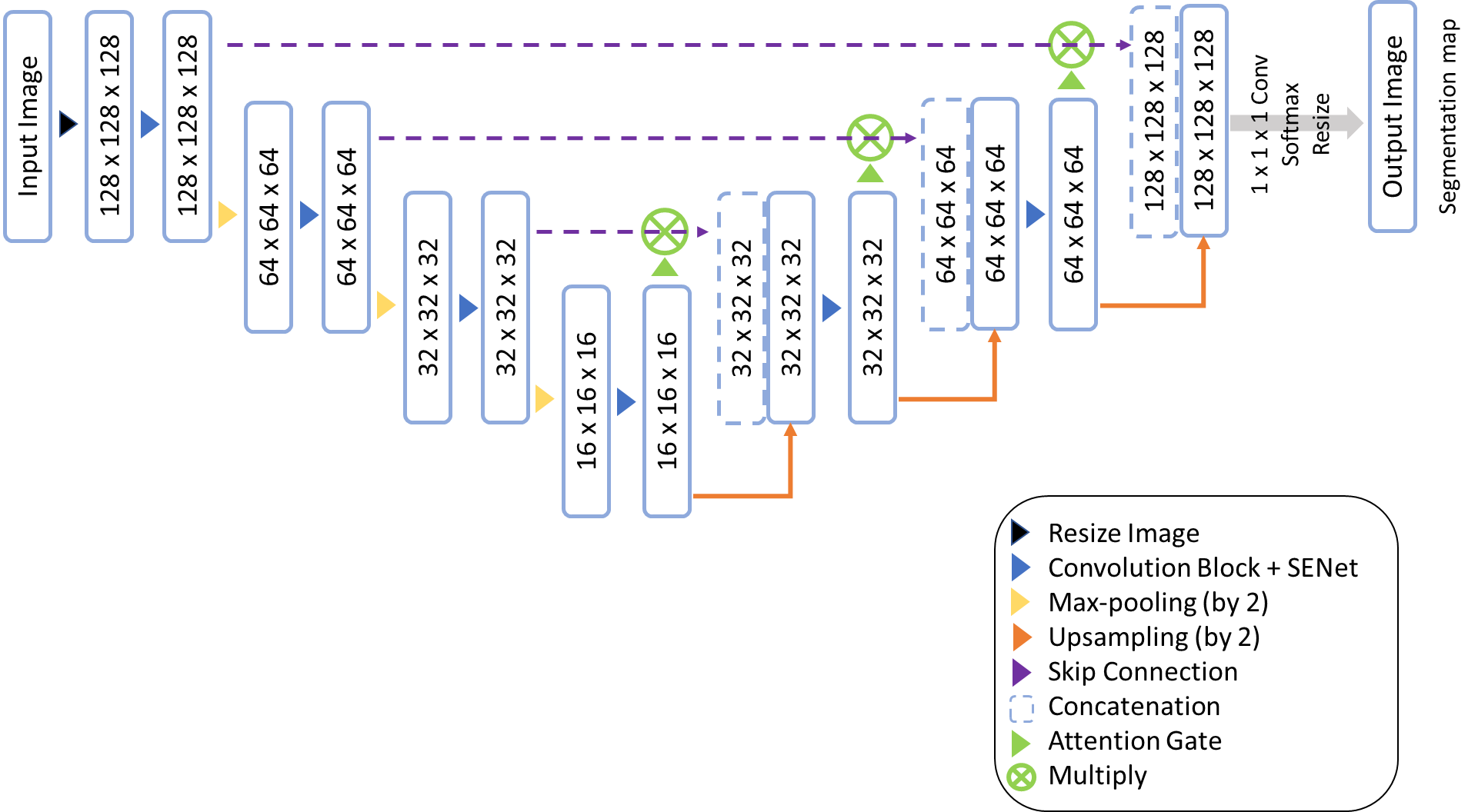


The figure shows the structure of deep learning model for facial feature detector in our study. It is based on U-net structure with attention gate, and input image is resized because the memory capacity occupied by the 3-dimensional Magnetic Resonance image is large.

The Python code for this model structure has released on GitHub. [https://github.com/yeonuk-Jeong/Defacer]
