# Supplementary material for: De-Identification of Facial Features in Magnetic Resonance Images: Software Development Using Deep Learning Technology
Source: J Med Internet Res. 2020 Dec 10;22(12):e22739. doi: 10.2196/22739 (PMC7759440; doi:10.2196/22739)
Supplement: Multimedia Appendix 3 [file jmir_v22i12e22739_app3.docx]

**DICOM header with personal information**

The DICOM standard includes header information, some of which may reveal personal information. We anonymized the DICOM image by deleting the 20 headers information below.

- (0008, 0012) Instance Creation Date
- (0008, 0013) Instance Creation Time
- (0008, 0020) Study Date
- (0008, 0030) Study Time
- (0008, 0021) Series Date
- (0008, 0031) Series Time
- (0008, 0022) Acquisition Date
- (0008, 0032) Acquisition Time
- (0008, 0023) Content Date
- (0008, 0033) Content Time
- (0008, 0080) Institution Name
- (0008, 0081) Institution Address
- (0008, 0090) Referring Physician's Name
- (0008, 1050) Performing Physician's Name Attribute
- (0008, 1070) Operators' Name
- (0010, 0010) Patient's Name
- (0010, 0020) Patient ID
- (0010, 0030) Patient's Birth Date
- (0010, 0040) Patient's Sex
- (0010, 1010) Patient's Age
