# Supplementary material for: De-Identification of Facial Features in Magnetic Resonance Images: Software Development Using Deep Learning Technology
Source: J Med Internet Res. 2020 Dec 10;22(12):e22739. doi: 10.2196/22739 (PMC7759440; doi:10.2196/22739)
Supplement: Multimedia Appendix 4 [file jmir_v22i12e22739_app4.docx]

**Face recognition test**


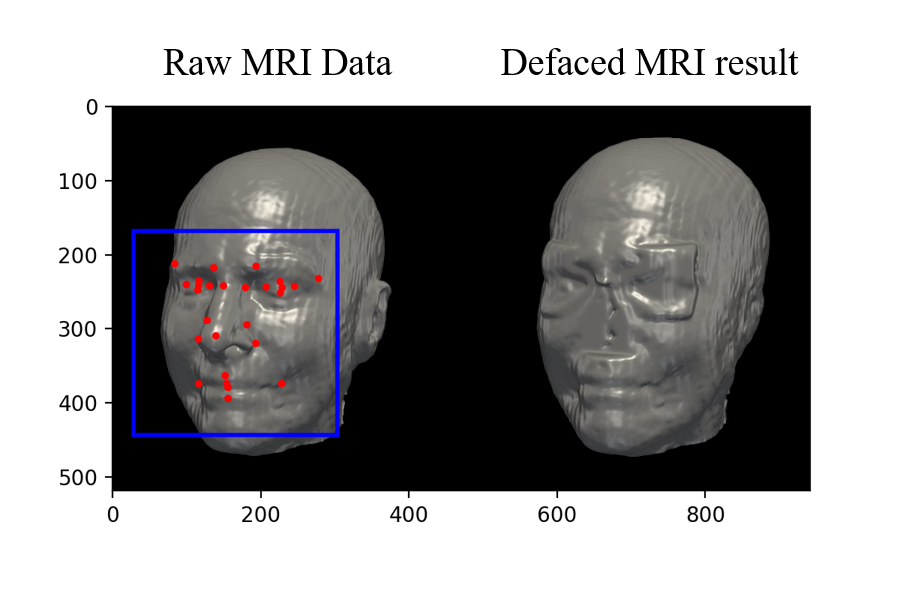


| **Face Rectangle** | **top** | **left** | **width** | **Height** |
| --- | --- | --- | --- | --- |
| **coordinate** | 239 | 110 | 325 | 325 |

| **Landmarks** | **x-coordinate** | **y- coordinate** |
| --- | --- | --- |
|  |  |  |
| pupilLeft | 115.8 | 241.9 |
| pupilRight | 228.7 | 244.4 |
| noseTip | 139.2 | 309.4 |
| mouthLeft | 115.9 | 374.2 |
| mouthRight | 228.1 | 374 |
| eyebrowLeftOuter | 83.6 | 212.1 |
| eyebrowLeftInner | 136.3 | 217.5 |
| eyeLeftOuter | 99.2 | 240.2 |
| eyeLeftTop | 116.4 | 235 |
| eyeLeftBottom | 114.8 | 247.4 |
| eyeLeftInner | 130.7 | 241.9 |
| eyebrowRightInner | 193.3 | 215.5 |
| eyebrowRightOuter | 277.8 | 232.1 |
| eyeRightInner | 207 | 243.5 |
| eyeRightTop | 225.9 | 236.1 |
| eyeRightBottom | 226.3 | 251.4 |
| eyeRightOuter | 245.4 | 242.6 |
| noseRootLeft | 149.4 | 241.9 |
| noseRootRight | 179.1 | 244.3 |
| noseLeftAlarTop | 127.4 | 288.5 |
| noseRightAlarTop | 181.1 | 294.4 |
| noseLeftAlarOutTip | 115.6 | 313.9 |
| noseRightAlarOutTip | 193.1 | 319.6 |
| upperLipTop | 151.7 | 363.2 |
| upperLipBottom | 153.7 | 373.9 |
| underLipTop | 155.5 | 379.1 |
| underLipBottom | 155.6 | 393.9 |
